# Pneumococcal vaccine impacts on the population genomics of non-typeable *Haemophilus influenzae*

**DOI:** 10.1099/mgen.0.000209

**Published:** 2018-08-06

**Authors:** David Cleary, Vanessa Devine, Denise Morris, Karen Osman, Rebecca Gladstone, Stephen Bentley, Saul Faust, Stuart Clarke

**Affiliations:** ^1^​ Faculty of Medicine and Institute for Life Sciences, University of Southampton, Southampton, UK; ^2^​ NIHR Southampton Biomedical Research Centre, University Hospital Southampton Foundation NHS Trust, Southampton, UK; ^3^​ Northern Ireland Centre for Stratified Medicine and Clinical Translational Research Innovation Centre, Londonderry, UK; ^4^​ Pathogen Genomics, Wellcome Trust Sanger Institute, UK; ^5^​ NIHR Southampton Clinical Research Facility, University Hospital Southampton Foundation NHS Trust, Southampton, UK; ^6^​ Global Health Research Institute, University of Southampton, Southampton, UK

**Keywords:** non-typeable *Haemophilus influenzae* (NTHi), pneumococcal conjugate vaccines, *Streptococcus pnuemoniae*, PCV13

## Abstract

The implementation of pneumococcal conjugate vaccines (PCVs) has led to a decline in vaccine-type disease. However, there is evidence that the epidemiology of non-typeable *Haemophilus influenzae* (NTHi) carriage and disease can be altered as a consequence of PCV introduction. We explored the epidemiological shifts in NTHi carriage using whole genome sequencing over a 5-year period that included PCV13 replacement of PCV7 in the UK’s National Immunization Programme in 2010. Between 2008/09 and 2012/13 (October to March), nasopharyngeal swabs were taken from children <5 years of age. Significantly increased carriage post-PCV13 was observed and lineage-specific associations with *Streptococcus pneumoniae* were seen before but not after PCV13 introduction. NTHi were characterized into 11 discrete, temporally stable lineages, congruent with current knowledge regarding the clonality of NTHi. The increased carriage could not be linked to the expansion of a particular clone and different co-carriage dynamics were seen before PCV13 implementation when NTHi co-carried with vaccine serotype pneumococci. In summary, PCV13 introduction has been shown to have an indirect effect on NTHi epidemiology and there exists both negative and positive, distinct associations between pneumococci and NTHi. This should be considered when evaluating the impacts of pneumococcal vaccine design and policy.

## Data Summary

1. Genomic data are deposited in the European Nucleotide Archive (ENA) under project PRJEB23674 with accession numbers ERS2026205 to ERS2026469.

2. Genomes as used by De Chiara *et al*. [1] were downloaded from ftp://ftp.sanger.ac.uk/pub/project/pathogens/Haemophilus/influenzae/NT_strains/.

**Impact Statement**

Widespread use of pneumococcal conjugate vaccines (PCVs) has reduced the disease burden from *Streptococcus pneumoniae* and altered the epidemiology of circulating serotypes. There is evidence that an indirect effect has been the increased carriage of non-typeable *Haemophilus influenzae* (NTHi), an opportunistic pathogen capable of causing both invasive and non-invasive disease. In some regional studies this has also led to an increase in disease. Although not ubiquitous, this outcome has been seen with PCV vaccines rather than those with a *Haemophilus–*protein conjugate such as PCV10 (Synflorix), suggesting that the vaccine is driving a change in NTHi epidemiology through niche disruption rather than induced immunity. Here we show that an indirect effect of PCV13 implementation is an increase in carriage of NTHi in children at the time of vaccine introduction. In addition, we find that co-colonization of pneumococci and NTHi appears to be driven by whichever genomic lineage of the latter is present. One lineage for example was more likely to be isolated from the nasopharynx of children who also carried *S. pneumoniae*. Whilst carriage increased, overall, the high diversity and robust population structure of NTHi was stable over time, which we suggest has clear implications for designs of future vaccines.

## Introduction

Non-typeable *Haemophilus influenzae* (NTHi) is a Gram-negative bacterium commonly found in the human nasopharynx. Severe invasive infections such as meningitis and septicaemia caused by capsulated *H. influenzae*, in particular serotype b (Hib), have been reduced with the widespread use of specific conjugate vaccines [2]. The global mortality and morbidity from NTHi, which predominantly replaced serotype b in disease, is significant [3]. This includes the association with acute otitis media (AOM) [4] and in exacerbations of chronic lung conditions such as cystic fibrosis [5, 6] and chronic obstructive pulmonary disease [7]. NTHi is now also the leading cause of invasive *H. influenzae* disease. In 12 EU/EEA countries between 2007 and 2014, NTHi accounted for 78 % of the 8781 cases of invasive disease with the burden highest in infants and those ≥60 years of age [8]. The former increase was largely down to a concerning 6.2 % [95 % confidence interval (CI) 2.8–9.8 %] annual increase in neonatal disease notification [8].

There is evidence that the epidemiology of NTHi carriage and disease has changed as a consequence of introduction of pneumococcal conjugate vaccines (PCVs). This includes an increase in the proportional isolation of NTHi in children with AOM [9–11] and increased nasopharyngeal carriage in children who have received PCVs [12, 13] although this is not a ubiquitous phenomenon [14]. In the UK two PCVs have been introduced in the routine childhood immunization schedule, PCV7 (Prevenar 7 introduced in 2006) and PCV13 (Prevenar 13 introduced in 2010), that provide protection against capsular serotypes most commonly associated with invasive disease in high-income countries. Importantly, they are not conjugates of *Haemophilus* protein D unlike PHiD10, a 10-valent PCV used in some other countries. Therefore, any effects on NTHi seen in the UK may be considered indirect rather than induced protective immunity to NTHi.

A limited understanding of the genomic-level population structure of NTHi has hindered efforts to elucidate impacts of PCV vaccination on carriage and disease due to NTHi. Such studies would enable greater clarity regarding previous observations on NTHi co-occurrences with certain serotypes of *Streptococcus pneumoniae* [15]. This paucity of data is also important as the organism’s high degree of genetic diversity is hampering the development of effective vaccines [16].

Here we use whole-genome sequencing of NTHi nasopharyngeal carriage isolates recovered from children <5 years of age recruited across a 5-year period and show that (1) the NTHi population exists in clearly delineated, temporally stable lineages, (2) the introduction of PCV13 increased the carriage of NTHi, in the absence of apparent selection for distinct clones or lineages, and (3) specific pneumococcal–NTHi lineage co-carriage associations exist that warrant further exploration.

## Methods

### Bacterial isolates

Nasopharyngeal swabs (*n*=1569) were collected from children aged <5 years of age who attended outpatient clinics at University Hospital Southampton NHS Foundation Trust during five consecutive winters, October to March, 2008/9 to 2012/13. This study, designed to characterize *S. pneumoniae* carriage, has been described in detail elsewhere [17–19]. Informed consent was obtained before or after an outpatient appointment, but no specific outpatient clinic was targeted, and these were not children specifically presenting to outpatients as a consequent of respiratory infection. The participant was either the child attending the clinic or their sibling. Only one child per family was swabbed. Age was the primary exclusion criterion. Rayon-tipped Transwabs (Medical Wire) in charcoal Amies media were plated onto chocolate agar with bacitracin within 9 h of collection at the Health Protection Agency Southampton Laboratory (now part of Public Health England) between 2008/09 and 2011/12 and by technical staff in our research group during 2012/13. Presumptive *Haemophilus* were subcultured/purity picked onto Columbia agar with chocolate horse blood. Several colonies were inoculated into 3 ml 0.85 % saline to 0.5 McFarland units and then plated using a sterile swab onto blood agar base with X (haemin), V (NAD) and XV discs (Oxoid). Presumptive *H. influenzae* were determined by growth around XV discs only. Only one colony of *Haemophilus* per participant swab was selected for further analysis (*n*=275).

### DNA extraction and quantification

The original isolate was re-grown and genomic DNA was extracted from a sweep of NTHi colonies using a QIAmp DNA Mini kit (Qiagen) as per the manufacturer’s instructions. The concentration of genomic DNA was determined using Qubit 2.0 fluorometric quantification (Thermo-Fisher).

### Whole genome sequencing, assembly and annotation

Libraries were prepared using Nextera XT and sequenced using Illumina MiSeq, with V2 chemistry to generate 2×250 bp paired-end read data to a depth of approximately 100-fold coverage for each bacterial isolate. Paired-end reads were trimmed using trimmomatic v0.32 [20] and *de novo* assembled using SPAdes v3.10.1 [21]. Contiguous sequence orientation and gap filling to create scaffolds was undertaken using the assembly_improvement script from the Wellcome Trust Sanger Institute (https://github.com/sanger-pathogens/assembly_improvement). Annotation was done using Prokka v1.10 [22].

### MLST and identification of capsular loci

Read mapping for designation of multi-locus sequence types (MLSTs) was done using SRST2 v0.1.5 [23]. Confirmation of capsular status was done by *in silico* PCR in exonerate v2.2.0 (https://github.com/nathanweeks/exonerate) using previously published primers [24].

### Antibiotic resistance gene identification

Identification of antibiotic resistance determinants was done using Ariba [25] v2.10.1 using the Comprehensive Antibiotic Resistance Database v1.2.0 [26]. Identification of β-lactamase-negative ampicillin-resistance (BLNAR) due to mutations in *ftsl* was made using primers SSNF2 and KTGR2 as previously described [27] using *in silico* PCR with iPCRess [28]. The presence of acquired macrolide resistance genes was determined using a primer mismatch tolerance of 4, to detect *erm*(A), *erm*(B), *erm*(C), *erm*(F), *mef*(A) and *mef*(E) using primers described previously [29]. In addition, isolates were also screened for mutations in *rplV* (L22), the 50S ribosomal subunit protein L4, and 23S rRNA which had previously been associated with azithromycin resistance [30, 31]. Alignments were done in Seaview v4.5.4. Results were visualized using Phandango (http://phandango.net/) [32].

### *Haemophilus influenzae* genomes

Publicly available genomes as used by De Chiara *et al*. [1] were downloaded from ftp://ftp.sanger.ac.uk/pub/project/pathogens/Haemophilus/influenzae/NT_strains/. Accession numbers for each genome used are given in the Supplementary Data.

### Analysis of population structure

Core genome alignments were constructed using Parsnp from Harvest v1.2 [33] with -x to filter recombination. Here the minimum MUM anchor was set to 17 to force alignment of more dissimilar genomes. The resultant xmfa was converted to fasta using the script xmfa2fasta.pl (https://github.com/kjolley/seq_scripts/blob/master/xmfa2fasta.pl), and Gblocks v0.91b [34] was used to remove contiguous non-conserved regions from the alignment. A maximum-likelihood (ML) phylogeny was reconstructed using the CIPRES-hosted RAxML-HPC v8 [35, 36] with a General Time Reversible model, GAMMA substitution rate and rapid bootstrapping (*n*=1000). Population structure was determined using hierBAPS [37] implemented with four hierarchy levels and an upper cluster limit of 20. The resultant tree and metadata were visualized using Microreact (https://microreact.org/) [38].

### Recombination

Recombination in the core genome was determined using ClonalFrameML [39] v1.11 with -emsim set at 100 simulations. The best ML tree, as produced by RAxML, was used as the starting tree. Estimates of the ratio of recombination to mutation (*R*/θ), the mean length of recombination imports (δ) and the average divergence of imports (*ν*) were used to determine *r*/*m* – the relative effect of recombination to mutation.

### Statistical analyses

All statistical analyses were done in R Studio v3.4.0. Carriage of NTHi was first tested independently against participant age, year of the study and carriage of *S. pneumoniae*. Additionally, association between age and study year was done using a linear model and tested for significance using both parametric (ANOVA) and non-parametric (Kruskal–Wallis and Fligner–Killeen) tests where the former tests for variance in means and assumes normal distribution and equal variance, in contrast to the latter non-parametric tests that are robust to non-normality of data distribution. Consequently, the final model was a generalized linear logistic model of NTHi carriage against both year of study and pneumococcal carriage [using the function *glm*() with ‘family=binomial’] where the comparator was the first year of the study and the analysis was controlled for by age. Multiple comparison correction of model outcomes was done using the function *glht*(linfct=mcp(’Tukey’)) from the package multcomp v1.4.8. *P* values of NTHi lineage associations with pneumococci were false discovery rate (FDR)-adjusted using the Benjamini–Hochberg procedure with an FDR of 25 %. Bray–Curtis dissimilarity was calculated using *vegdist*(method=‘bray’) in the R package Vegan [40]. Simpson's (1−*D*) index was calculated using the formula 1−(Σ*n*(*n−*1)/*N*(*N*−1)) where *n* was the number of each MLST and *N* the total number of MLSTs.

### Genomic data

Data are deposited in the European Nucleotide Archive (ENA) under project PRJEB23674 with accession numbers ERS2026205 to ERS2026469.

## Results

### Introduction of PCV13 in the UK increased carriage of NTHi in children <5 years of age

Between the winters of 2008/09 and 2012/13 a total of 1569 nasopharyngeal swabs were taken from children <5 years of age attending outpatient clinics at a large UK hospital, University Hospital Southampton NHS Foundation Trust (NHS Research Ethics 06/Q1704/105). Of 275 *Haemophilus* isolates recovered (Table 1), 99.3 % (*n*=273) were classified as NTHi using *in silico* capsular analysis. Three of these were *bexB*/*bexA*^+/−^ and therefore classifiable as capsular-deficient [24]. The two non-NTHi isolates were serotype f.

**Table 1.** Carriage of non-typeable *Haemophilus influenzae* (NTHi) and *Streptococcus pneumoniae* (Spn) with age distribution of participants NTHi carriage was significantly increased (*^±^*P*<0.05) in 2010/11. No change in *S. pneumoniae* carriage was observed. The age of participants was significantly higher (***^β^*P*<0.001) in 2008/09 compared to all other years except for children who carried NTHi in 2011/12. Between children who carried NTHi, age was significantly increased (***^δ^*P*<0.001) in 2010/11 and 2011/12 compared to 2009/10 and 2012/13. **^ϕ^The average age of NTHi carriers was significantly higher (*P*=0.01) than that of all participants.

**Study year**	**2008/9**	**2009/10**	**2010/11**	**2011/12**	**2012/13**	**Mean**
Participants (*n*)	328	399	287	332	223	314 (310.8–317.2)‡
Bacterial carriage (%)						
NTHi	14.63 (*n*=48)	14.79 (*n*=59)	22.65 (*n*=65)^*±^	16.57 (*n*=55)	21.52 (*n*=48)	18.03 (17.58–18.48)‡
Spn	31.10 (*n*=102)	27.82 (*n*=111)	34.84 (*n*=100)	29.73 (*n*=99)	34.53 (*n*=77)	31.6 (31.33–31.87)‡
Co-colonized (NTHi+Spn)	6.71 (*n*=22)	5.76 (*n*=23)	8.71 (*n*=25)	7.23 (*n*=24)	10.76 (*n*=24)	7.83 (7.48–8.18)‡
% Participants (*n*) by age†						
0–2	0.61 (2)	18.80 (75)	7.32 (21)	5.42 (18)	2.24 (5)	7.64 (24)
3–12	25.61 (84)	33.83 (135)	34.15 (98)	33.43 (111)	45.29 (101)	33.76 (106)
13–24	26.52 (87)	25.31 (101)	25.78 (74)	30.72 (102)	31.39 (70)	27.71 (87)
25+	44.82 (147)	21.05 (84)	30.31 (87)	28.92 (96)	16.59 (37)	28.66 (90)
% NTHi carriers (*n*) by age†						
0–2	0.00 (0)	6.78 (4)	3.08 (2)	1.82 (1)	0.00 (0)	01.82 (1)
3–12	10.42 (5)	32.20 (19)	26.15 (17)	20.00 (11)	43.75 (21)	27.27 (15)
13–24	27.08 (13)	27.12 (16)	36.92 (24)	30.91 (17)	37.50 (18)	32.73 (18)
25+	60.42 (29)	33.90 (20)	33.85 (22)	49.09 (27)	16.67 (8)	38.18 (21)
Average age of all participants (months)	25^***β^	15	20	20	16	19**^ϕ^ (18.80–19.20)‡
Average age of NTHi carriers (months)	31^***β^	19	22^***δ^	26^***δ^	18	23**^ϕ^ (22.34–23.633)‡

†Age records for 37 participants were not available.

‡95 % CIs are shown in parentheses.

**Fig. 1.**
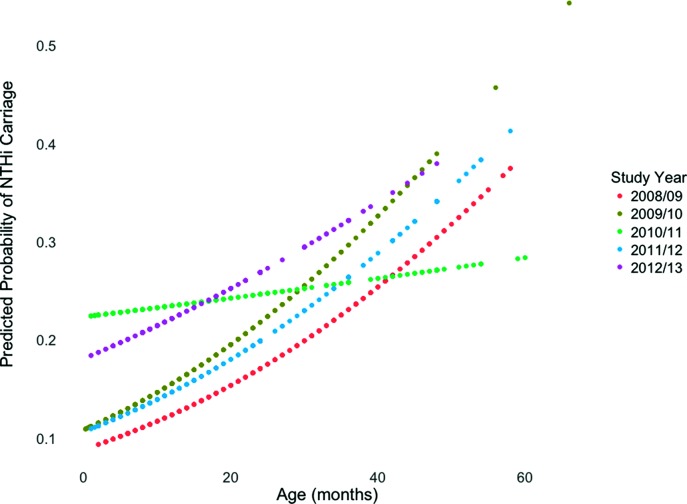
Predicted probabilities for NTHi carriage against increasing age (in months) for each of the study years. 2010/11 was the period where PCV13 was being introduced and the clear change in relationship between increasing age and increased odds coincides with an increased carriage prevalence in our study cohort.

Carriage prevalence ranged from 14.6 % in 2008/9 to 22.7 % in 2010/11 with a mean of 18.0 % (95 % CI 17.58–18.48 %) (Table 1). Linear regression with both parametric and non-parametric tests showed significant differences in the age of participants across the study period (*P*<0.001) with those recruited in 2008/09, the first year of the study presented here, being an older population compared to all other years. After adjusting for age, a multivariable, binomial logistic regression model revealed a higher carriage prevalence of NTHi in 2010/11, the period of PCV13 introduction (*P*<0.05). Overall, children who carried NTHi were older (*P*<0.05) (Table 1) and every increase in age by 1 month was significantly associated with increased odds of carriage [odds ratio (OR) 1.02, *P*<0.001]. In 2010/11 there was a clear alteration in the relationship of age and predicted probability of NTHi carriage in contrast to the other four years examined (Fig. 1).

Presence of *S. pneumoniae* was significantly associated with NTHi carriage (*P*<0.001, OR 1.92, 95 % CI 1.66–2.17), with co-carriage varying from 5.8 % in 2009/10 to 10.8 % in 2012/13, although this difference was not significant. Comparing NTHi co-carriage with *S. pneumoniae* vaccine serotype (VT) versus non-VT in 2008/09 and 2009/10 (the years preceding PCV13 introduction) revealed significantly increased odds of co-carrying VT pneumococci (*P*<0.05, OR 2.36, 95 % CI 1.17–4.75). The three most common co-carried VT serotypes were 19A (*n*=19), 6A (*n*=11) and 6B (*n*=6). In 2011/12 and 2012/13, the post-PCV13 period, six instances of VT co-carriage were seen, three involving serotype 3 and one each of 6A, 5 and 19A. Of all the 26 pneumococcal serotypes identified both with and without NTHi co-carriage between 2011 and 2013, 20 were found to be co-carried with NTHi. The most common serotypes co-carried were 11A, 23A and 15B, which accounted for 68 % of all cases of co-carriage. These were the 3rd, 7th and 4th most prevalent serotypes over this 2-year period, respectively [17]. Interestingly the two most common pneumococcal serotypes found in this period, 15A and 23B, were each found in one case of co-carriage only.

**Fig. 2.**
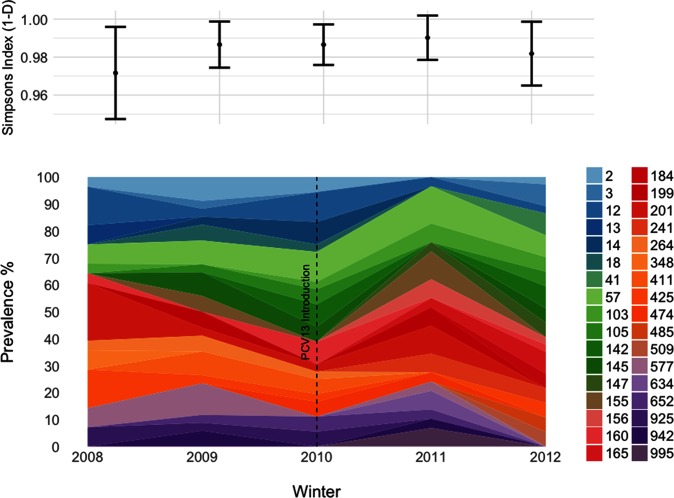
NTHi MLST prevalence and diversity during PCV implementation. For visual clarity, MLST percentage prevalence (bottom) is only shown for sequence types that were identified at least twice in any given year of the study. The dashed line denotes the period of PCV13 introduction. Simpson's 1−*D* is shown above for each year of the study; error bars are 95 % confidence intervals.

In total, 119 individual NTHi MLSTs were identified (Fig. 2). MLST diversity, as measured using Simpson's index (1−*D*), within each year was high, ranging from 0.97 in 2008/09 to 0.99 in 2011/12 (Fig. 2). No statistically significant increases in MLST diversity were noted during this period.

### The NTHi population can be defined by 11 discrete and temporally stable lineages

**Fig. 3.**
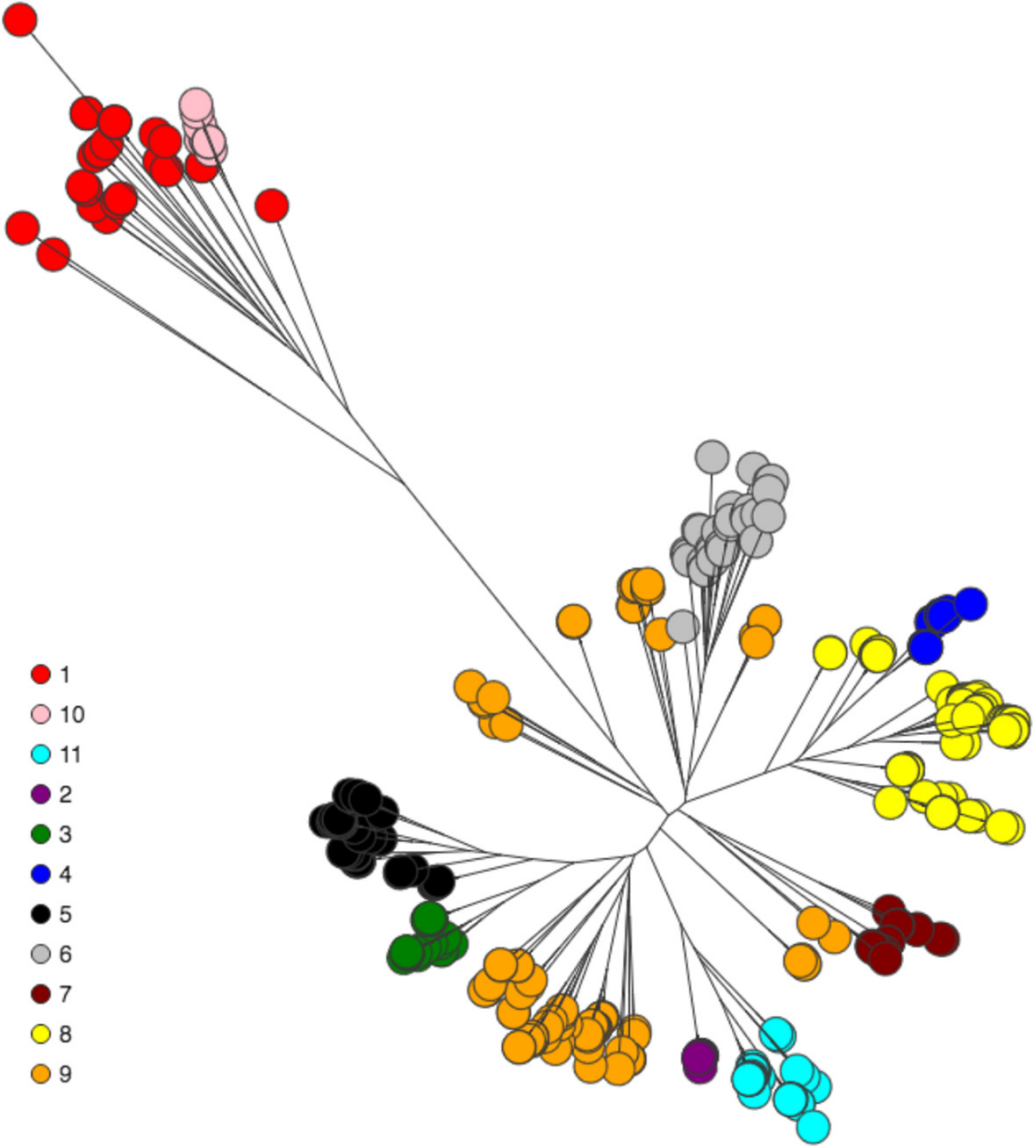
Population structure of the NTHi carriage isolates as determined by hierBAPS analysis. Terminal nodes of the ML core genome tree are coloured according the 11 lineages identified.

Fig. 3 shows the population structure for 265 NTHi carriage isolates for which genomic data were available in combination with an additional 89 isolates which had previously been used to identify six phylogenetic clusters [1]. Those excluded from the initial 275 had a sequencing depth of less than 30-fold coverage (the mean depth for all isolates was 106-fold, range=24–341) or assembled into >300 contigs (mean for all isolates=61.8, range=12–1179). The total core genome alignment length represented only 313 kbp (16.3 %) of the on average 1.92 Mbp genome. hierBAPS analysis revealed 11 lineages of NTHi. The polyphyletic Lineage 9 was the most predominant (*n*=54; 20.1 % of the carriage isolates). In reality this represents a collection of rarer lineages and their grouping does not suggest a common ancestor. For completeness it was included in subsequent analyses but not focused upon. The next most common, and monophyletic lineage, was 8 (*n*=47; 17.7 %) with lineage 2 containing the fewest isolates (*n*=5). All 89 additional isolates were classified concordantly with their six previously identified clusters, but with additional delineation. Isolates belonging to cluster IV were further separated into lineages 2 and 11, cluster V into lineages 8 and 4, and cluster VI into lineages 6, 7 or 9 (Supplementary Data).

**Fig. 4.**
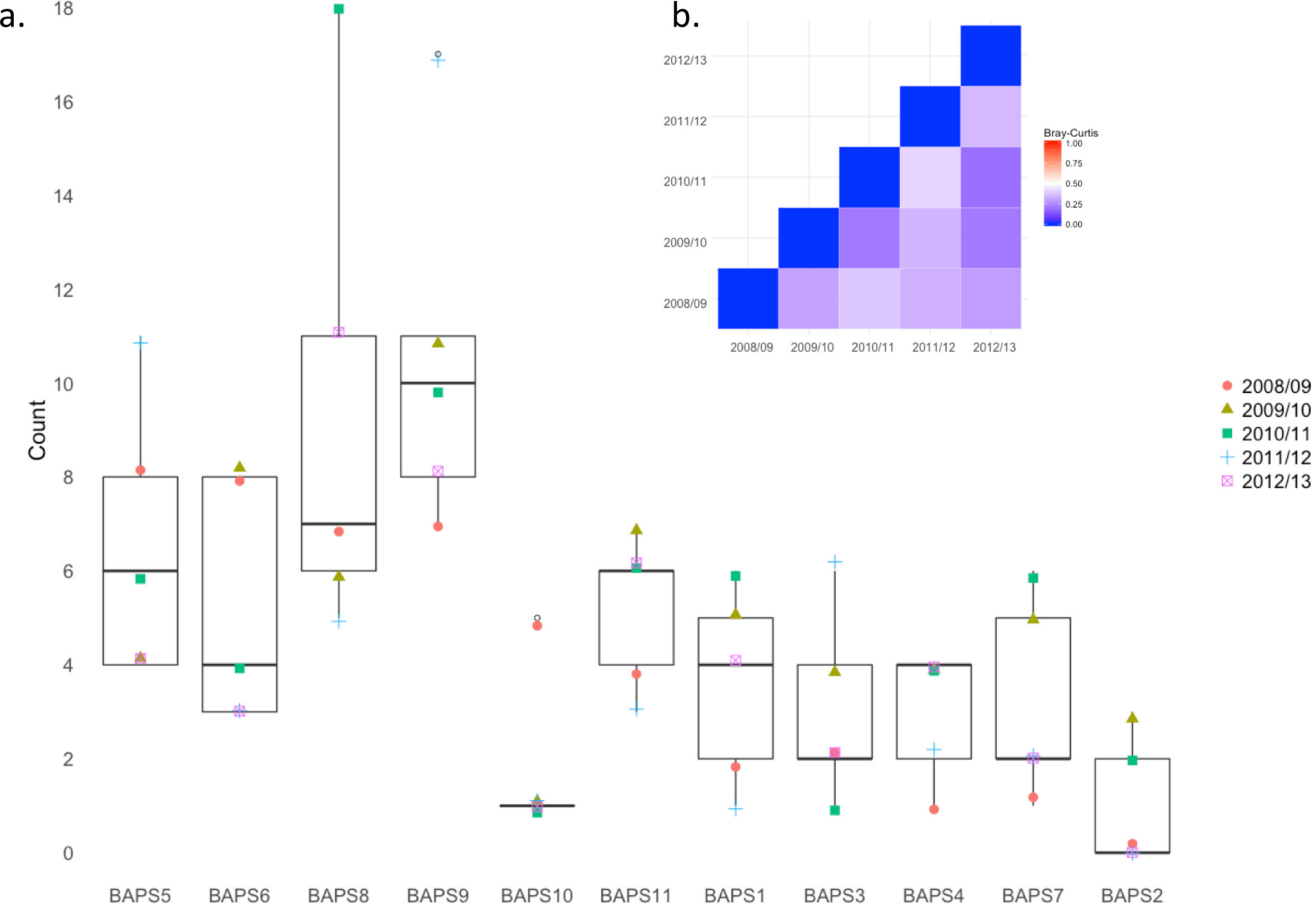
(a) Box and whisker plot showing NTHi hierBAPS lineage occurrence across years. Lineages are ranked according to the abundance in 2008/09. Coloured symbols represent each of the five sampling periods 2008/09 to 20012/13. (b) Heat map showing Bray–Curtis dissimilarity index based on presence and abundance of hierBAPS lineages within each year of the study. Red indicates less similar populations and blue highly similar structuring.

We sought to address the paradox of an apparently highly diverse collection of NTHi, based on number of sequence types identified, within relatively few lineages. We hypothesized that the intra-lineage diversity is balanced by a stable population structure at a higher level of clustering. Here Bray–Curtis dissimilarity was used to account for both lineage presence and abundance between years. As shown in Fig. 4(a), lineages exhibited very little difference between years. Any variation was not due to PCV13 introduction as the most dissimilar years were both post-PCV13 introduction, 2010/11 and 2011/12 (0.41 Bray–Curtis dissimilarity index) (Fig. 4b).

MLST diversity within lineages varied considerably (Fig. S1) ranging from 17 unique MLSTs in the monophyletic lineage 8 to lineage 2, which consisted of just five isolates of ST411. The most common sequence types encountered were ST57 (*n*=15, lineage 11), ST201 (*n*=11, lineage 5) and ST12 (*n*=11, lineage 8). Lineage prevalence was not a good predictor of intra-lineage diversity as measured by Simpson's 1−*D*, *r*[10]=0.122, *P*>0.5.

### NTHi lineage 6 was significantly associated with carriage of *S. pneumoniae* pre-PCV13

**Fig. 5.**
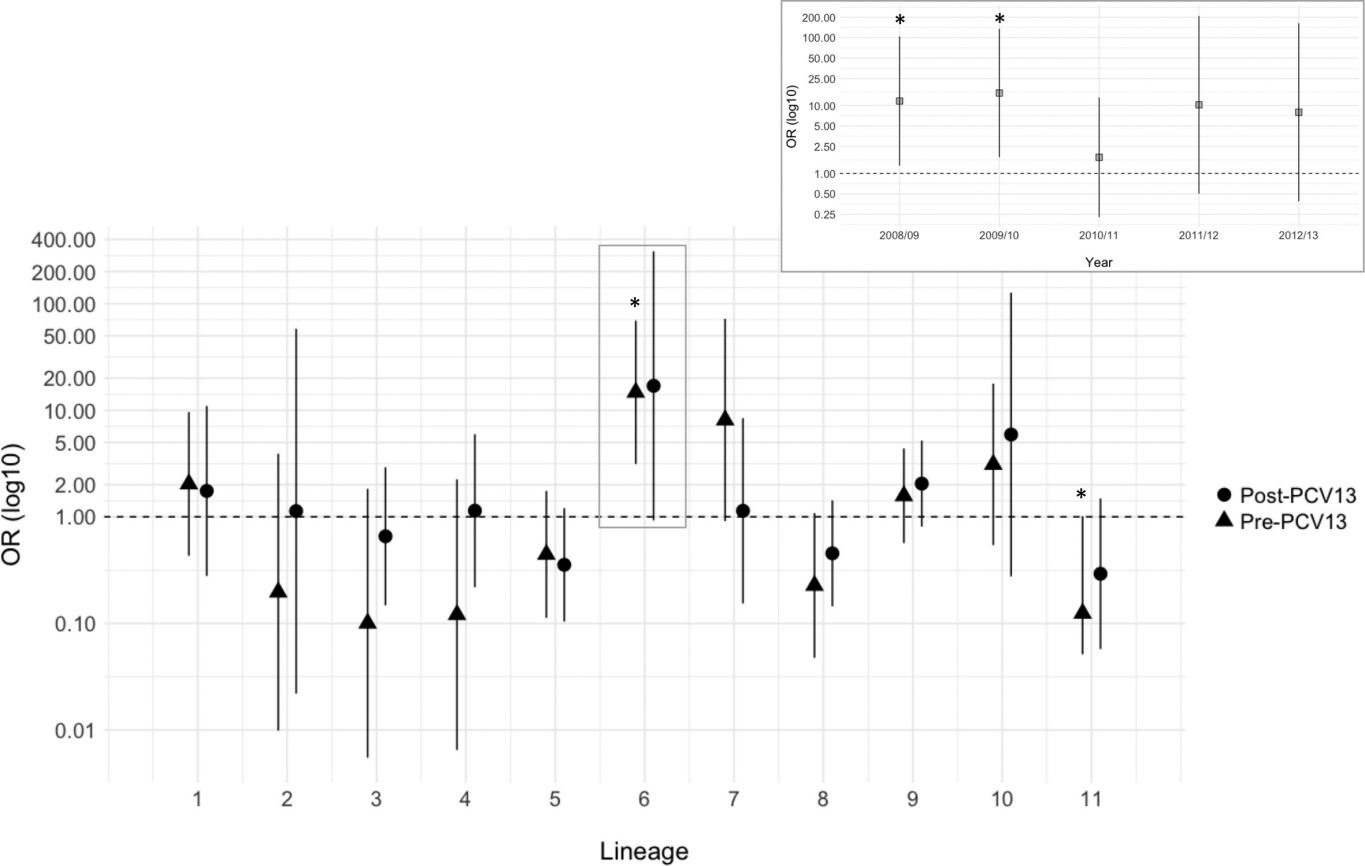
Odds of co-carriage of *S. pneumoniae* for each NTHi lineage pre- and post-introduction of PCV13. Here ORs were calculated using data from 2008/09 to 2009/10 as pre-PCV13 and 2011/12 to 2012/13 as post-PCV13. Lineage 6 pre-PCV13 was significantly (*P<0.05) associated with carriage of *S. pneumoniae*. In the same period lineage 11 was significantly (*P<0.05) associated with non-carriage of *S. pneumoniae*. These two associations were not present in the post-PCV13 era. Inset is shown the OR for co-carriage of *S. pneumoniae* for lineage 6 across all years.

The odds of isolating a lineage of NTHi co-carried with *S. pneumoniae* did not change markedly following PCV13 introduction (Fig. 5). However, the likelihood of co-carriage did vary between lineages. Lineage 6 had an OR for co-carriage of 14.75 (95 % CI: 3.14–69.38) and 16.95 (95 % CI: 0.93–309.96) pre- and post-PCV13, respectively, with the former being statistically significant (*P*<0·05); co-carriage was found for 87.5 and 100 % of isolates, respectively. Interestingly these increased odds were significant in the two years prior to PCV13 until 2010/11, when we predict the impact of PCV13 may have begun, whereupon this relationship was altered (Fig. 5). Pre-PCV13, 31.3 % (5/18) of lineage 6 isolates were co-carried with vaccine serotype pneumococci (serotypes 6B, 19A and 7F). In comparison only eight lineage 6 isolates were found in the following three post-PCV13 years, none of which was VT. The absence of VT pneumococci in this period coupled with a lower isolate number leaves this potential impact of PCV13 open to interpretation. By contrast, in the pre-PCV13 era lineage 11 exhibited a negative association with *S. pneumoniae* (OR 0.12, 95 % CI: 0.05–1.01, *P*<0.05), although this was not statistically significant in the post-PCV13 era.

### Diverse levels of recombination exist between NTHi lineages

Within the lineages an almost universally high degree of recombination was observed (Table S1). Although recombination occurred on average 25 % less often than mutation (ratio of recombination to mutation: *R*/θ 0.742, 95 % CI: 0.713–0.771), it produced 19 times (relative effect of recombination to mutation: *r*/*m* 19.075, 95 % CI: 17.38–20.77) more substitutions than *de novo* mutation. Exceptions included lineages 1, 7 and 9 which had significantly lower relative impacts of recombination with *r*/*m* values of 3.467 (*P*=0.0002), 10.642 (*P*=0.0267) and 4.651 (*P*=0.0005), respectively. In addition, lineage 10 had an *R*/θ of 1.103, indicating recombination occurred approximately 10 % more often than mutation and this caused 56 times more substitutions. MLST diversity within each lineage was correlated to *r*/*m* such that lineages with lower Simpson's 1−*D* (and thus lower diversity) were shown to have higher *r*/*m* (*r*[10] 0.733, *P*<0.001).

### Recombination hot-spots are characterized by involvement in metabolic/biosynthetic pathways

**Fig. 6.**
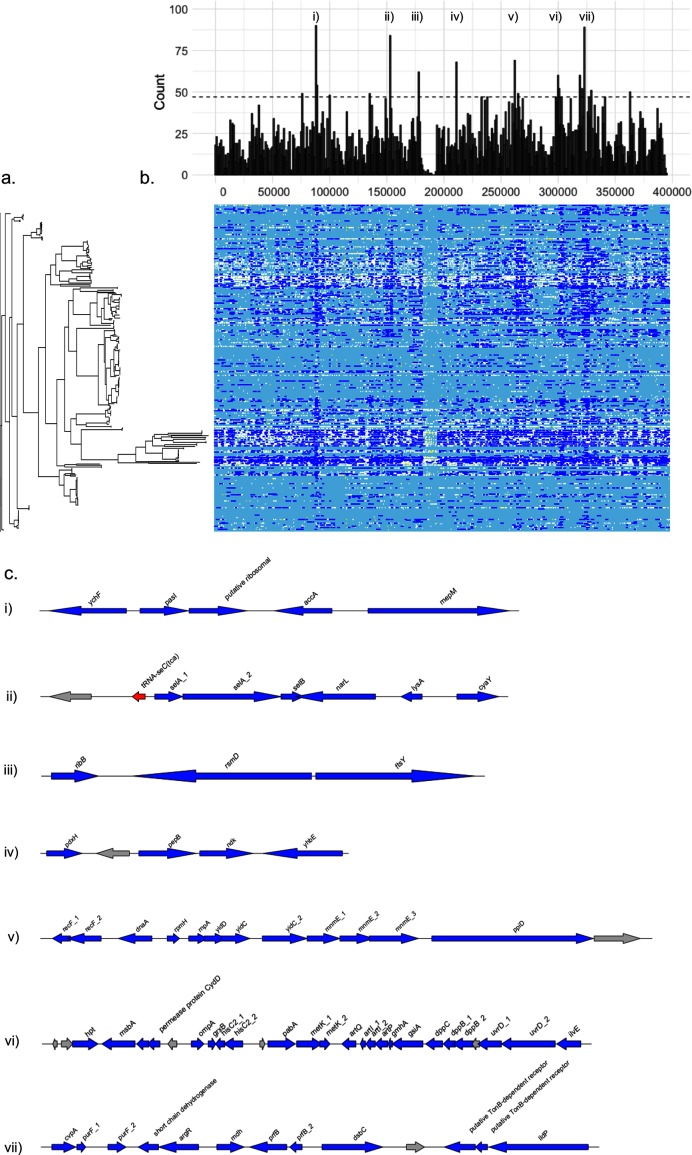
Recombination in the core genome of NTHi. ML phylogeny and a recombination plot using ClonalFrameML (a) is shown with a density plot of recombination blocks across the core genome alignment (b). Here seven regions that had a density of recombination blocks above the 95th quantile are shown. Annotations are shown in (c), with grey arrows denoting hypothetical proteins, red denoting tRNAs and blue denoting identifiable coding genes.

Recombination across the core genome is shown in Fig. 6(a). Here recombination is shown by dark blue and mutation by white. The variability between lineages is clear, as is the non-uniformity of recombination across the core genome alignment. This variability is illustrated in Fig. 6(b) where recombination blocks are counted and plotted according to position in the alignment.

Seven regions were shown to contain recombination counts above the 95 % quantile. Annotations of these are shown in Fig. 6(c). The predominant characteristic of these loci is the presence of genes involved in metabolic and biosynthesis pathways. These include selenoprotein biosynthesis (*selA*1-2, *selB*), amino acid metabolism and scavenging (*pepB*, *ilvE*, *hisC2*, *grxB*, *art*, *hpt*, *cydD*), carbon source utilization (*metK*1/2, *lidP*), and protein formation and transport (*dsbC*, *yhbE*, *msbA*, *yidD*/*C*, *ppiD*). Several genes that play roles in responses to nutrient availability/stress were also noted and included *narL* which mediates nitrate-response transcriptional regulation [41], the DNA repair-associated genes *recF1* and *recF2*, and adenylate cyclase (*cyaD*) which controls competence. Of note is that only one gene associated with outer membrane proteins was identified, *ompA*, which encodes outer membrane protein P5.

### Prevalence of antibiotic resistance genes is low except for BLNAR mutations in ST411 of lineage 2

**Fig. 7.**
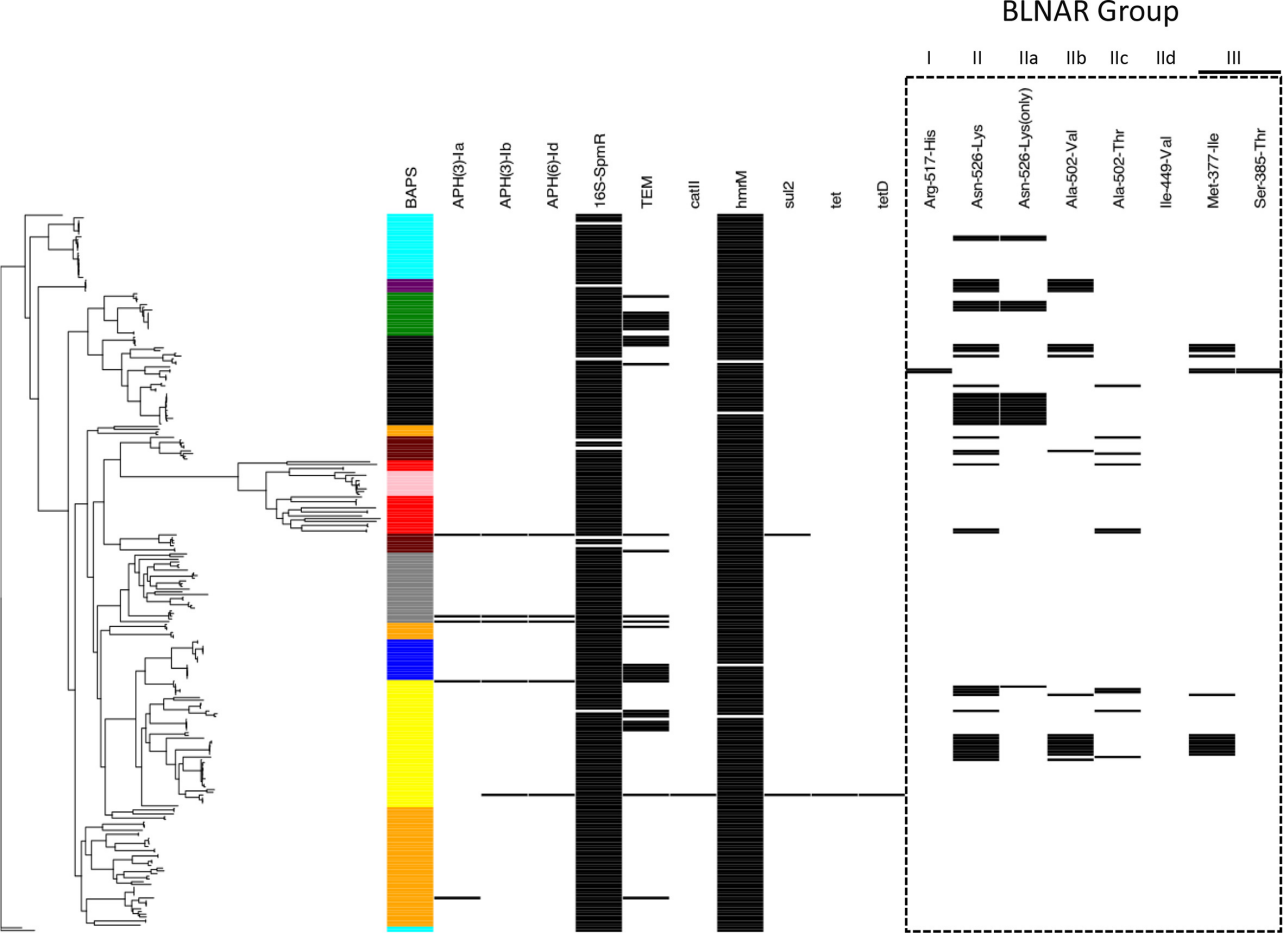
Identification of antimicrobial resistance genes and mutations in NTHi. The ML phylogeny (left) is annotated by coloured block according to hierBAPS lineage designation. The presence of loci/mutations associated with resistance is shown in black. Mutations in the Penicillin Binding Protein 3 (PBP3) encoding *ftsl*, associated with BLNAR, are highlighted by the dashed rectangle.

The identification of antibiotic resistance genes is shown in Fig. 7. Alleles for spectinomycin resistance and the multi-drug efflux pump, *hmrM*, were found to be near ubiquitous at 97.0 % (*n*=257) and 98.9 % (*n*=262) of isolates, respectively. APH (Aminoglycoside O-Phosphotransferase) [4] alleles for aminoglycoside resistance were found in fewer than 1.9 % (*n*=5) of isolates which included sequence types from lineages 6 (ST932 and 264), 7 (ST154), 8 (ST3 and 142) and 9 (ST1411). One of these isolates (a lineage 8, ST3 from 2012/13) was also positive for *catII* (chloramphenicol), *tet* and *tetD*. The same isolate carried resistance to sulphonamide (*sul2*) as did one other, a lineage 7, ST154 from 2008/09. Of the 34 isolates in which β-lactamase resistance (TEM) was identified, seven belonged to lineage 3 (ST577 *n*=6, ST156 *n*=1), six to lineage 4 (ST103), five to lineage 5 (ST165 *n*=4) and two to each of lineages 6, 7 and 9. The majority (*n*=9/34), however, were from lineage 8, four of which were ST160. TEM subtypes numbered 19 in total, with TEM-122, TEM-176, TEM-186 and TEM-206 being the most common (*n*=14/34).

No acquired macrolide resistance genes were identified. Of the mutations in L4 and L22 previously reported in isolates with high-level macrolide resistance [30], only K61Q in L4 and the RAKG insertion at position 88 in L22 were found. However, in both cases these were ubiquitous, which questions whether the association previously identified was in fact causative. Lastly, none of the 23S rRNA mutations at positions 2058, 2059 or 2611 (based on *Escherichia coli* numbering) were found.

The presence of mutations in *ftsl*, encoding the transmembrane component of penicillin binding protein (PBP) 3, which results in BLNAR, was also determined [42]. Profiling of these gives rise to three groupings based on the mutations: Group I (His for Arg at 517), Group II (Lys for Asn at 526) and Group III (Ile for Met at 377, Phe for Leu at 389 or Thr for Ser at 385). Group II isolates can be subtyped into a further four profiles, a to d. Group IIa has the defining Asn-526-Lys without a substitution for Ala-502, IIb has a Ala-502-Val, IIc Ala-502-Thr and finally IId Ile-449-Val. Intermediate resistance is associated with Groups I and II whereas higher levels of resistance are more common with Group III [43]. No significant difference in the level of BLNAR observed in each year was found using a pairwise comparison of proportions test.

BLNAR mutations were found in 19.2 % (*n*=51) of isolates from seven lineages (Fig. 7). Only two Group I were identified, both belonging to ST155 of lineage 5. The Group II intermediate-BLNAR genotypes were almost ubiquitous in the BLNAR isolates accounting for 96.1 % (*n*=49). Here Groups IIa, IIb and IIc accounted for 38.8, 40.8 and 20.4 %, respectively. No Group IId was observed. The mutations that have previously been characterized as producing high-level, Group III, resistance [27] were found in 8.3 % of isolates (*n*=22) of which 20 also harboured Group II mutations and the remaining two Group I. The majority of the Group IIIs were restricted to lineages 8 (*n*=10) and 5 (*n*=6). Interestingly all lineage 2 isolates (ST411, *n*=5) harboured the Ala-502-Val mutation.

## Discussion

Non-typeable strains of *Haemophilus* are recognized to cause significant human disease including community-acquired pneumonia [44]. We explored epidemiological shifts in NTHi carriage over a 5-year period that included PCV13 replacement of PCV7 in the UK’s National Immunisation Programme in 2010. We demonstrate increased carriage of NTHi in children <5 years of age following this time point, which was not linked to the expansion of any one particular clone. To our knowledge this is the first study to demonstrate the temporal stability of NTHi lineages pre- and post-introduction of a PCV. We have shown the existence of a discrete lineage structure that is stable over time and that did not measurably shift in response to PCV13. The clonality of the population was concordant with previous examinations of population structure based on MLSTs [45] and whole genomes of geographically diverse isolate collections [1]. The hierarchical analysis we used gave greater phylogenetic clarity and revealed hitherto unrecognized resolution of the lineages of NTHi.

We also observed that in the pre-PCV13 era, NTHi carriage was more associated with carried pneumococcal serotypes that would ultimately be targeted by PCV13 (19A, 6 and 11) compared to non-VT strains. Data from previous studies have also shown that PCV13 serotypes were more associated with *Haemophilus* carriage in contrast to non-PCV13 serotypes [15]. This suggests there may be serotype-dependent carriage dynamics between these bacterial species. If this is the case, then a plausible outcome of vaccination could be a PCV-induced disruption of these associations. We believe this is the first description of specific NTHi lineages that exhibit potential interactions with pneumococci, regardless of whether these are VT serotypes. The fact that there could be NTHi strains that differ in the direction and amplitude of interaction with pneumococci is not surprising as there are many examples of competition, antagonism and synergism between the two genera [46–49] suggestive of a complex relationship. Specifically, co-carriage likelihoods per lineage were not consistent with lineage 6 associated with an increased likelihood of pneumococcal carriage and the converse for lineage 11. We recognize that many more isolates would be required to confirm and then examine the basis of these interactions; lineage 6, for example, represented less than 10 % of the NTHi isolates collected in this study. These low numbers render the application of genome-wide association studies virtually impossible. In addition, the phenotypes are not clearly delineating (as is, for example, antibiotic resistance) and they reside within a monophyletic lineage that poses a particular challenge to association studies of this nature (although methods to account for this within bacterial populations do exist) [50].

Recombination varied considerably in this population and correlated to intra-lineage diversity. Recombination has previously been shown to be a significant driver of *Haemophilus* species genetic diversity [1, 45], modulation of outer-membrane protein diversity [51] and transfer of antibiotic-resistant genes [52]. The high levels of recombination observed here, in terms of both frequency and locus involvement, are in keeping with that observed by Cody *et al*., who found recombination in genes associated with lipopolysaccharide biosynthesis as well as in housekeeping genes [53].

There are a few limitations to this study. Firstly, the sampling is limited to one geographical region and a defined, narrow subset of the population (children <5 years of age). The extrapolation of the genomic population structure detailed in this study to that of NTHi more generally must therefore be done with caution, although similar low numbers of lineages have previously been identified from much broader isolate collections [1, 45, 54]. Lastly these isolates are all from carriage and may not be representative of the genotypic diversity associated with disease. It will be interesting for future work to compare the diversity between strains isolated from chronic and acute pathologies from both children and older age cohorts.

The use of PCVs has radically reduced the burden of invasive pneumococcal disease (IPD) both in the UK [55] and globally [56–62]. In the UK, this IPD reduction has occurred in the absence of any loss of overall pneumococcal carriage prevalence [17, 18], and is a consequence of serotype replacement. The lack of penetrance between carriage replacement with non-VT serotypes and IPD is a consequence of lower invasiveness in the non-VT pneumococci [63]. Regardless, the replacement of VT pneumococci represents a disruption in nasopharyngeal microflora. Evidence for indirect effects associated with PCV on *Haemophilus* disease and carriage has been noted [9–13] and PCV vaccination in the very young (<12 months) has been shown to cause a more disordered nasopharyngeal microbiota [64, 65]. We hypothesize that this niche disruption, in combination with the adaptation of *S. pneumoniae* to maintain its relative fitness in the face of selective pressures in response to PCV introduction (Red Queen dynamics) [66] impacted the interactions between pneumococci and other microbiota resident in the nasopharynx. The disruption appears to manifest as the general, non-specific flux in carriage prevalence of NTHi (as the dominant *Haemophilus* species) seen here. The ramifications of there being particular lineages of NTHi that have extreme competitive or commensal relationships with pneumococci are important clinically in populations. Specifically, the impact of PCV on nasopharyngeal microbiomes will influence future conjugate vaccine design and use as well as interpretation of experimentally derived interaction models.

Through this analysis we have shown that the introduction of PCV13 probably influenced the epidemiology of NTHi by increasing carriage prevalence in a paediatric population. This included altered associations between specific genomic lineages of NTHi with pneumococci. Although more detailed in-depth study is needed, these initial findings indicate a benefit in examining these interactions and the potential implications for the implementation of future pneumococcal vaccinations as well as the design of any future *Haemophilus* vaccine.

## Data bibliography

1. Cleary DW, Devine VT, Morris D, Osman KL, Gladstone RA *et al*. Phylogenomics of NTHi during PCV13 Implementation PRJEB23674 (ERP105441) 2018.

## Supplementary Data

Supplementary File 1

Supplementary File 2
