# Corrigendum: Pneumococcal vaccine impacts on the population genomics of non-typeable *Haemophilus influenzae*

**DOI:** 10.1099/mgen.0.000559

**Published:** 2021-05-04

**Authors:** David W. Cleary, Vanessa T. Devine, Denise E. Morris, Karen L. Osman, Rebecca A. Gladstone, Stephen D. Bentley, Saul N. Faust, Stuart C. Clarke

**Affiliations:** ^1^​ Faculty of Medicine and Institute for Life Sciences, University of Southampton, Southampton, UK; ^2^​ NIHR Southampton Biomedical Research Centre, University Hospital Southampton Foundation NHS Trust, Southampton, UK; ^3^​ Northern Ireland Centre for Stratified Medicine and Clinical Translational Research Innovation Centre, Londonderry, UK; ^4^​ Pathogen Genomics, Wellcome Trust Sanger Institute, UK; ^5^​ NIHR Southampton Clinical Research Facility, University Hospital Southampton Foundation NHS Trust, Southampton, UK; ^6^​ Global Health Research Institute, University of Southampton, Southampton, UK

There was a change in the author names in the published article. The new list should read:

David W. Cleary^1,2^, Vanessa T. Devine^3^, Denise E. Morris^1^, Karen L. Osman^1^, Rebecca A. Gladstone^4^, Stephen D. Bentley^4^, Saul N. Faust^1,5^, Stuart C. Clarke^1,2,6^

^1^Faculty of Medicine and Institute for Life Sciences, University of Southampton, Southampton, UK.

^2^NIHR Southampton Biomedical Research Centre, University Hospital Southampton Foundation NHS Trust, Southampton, UK.

^3^Northern Ireland Centre for Stratified Medicine and Clinical Translational Research Innovation Centre, Londonderry, UK.

^4^Pathogen Genomics, Wellcome Trust Sanger Institute, UK.

^5^NIHR Southampton Clinical Research Facility, University Hospital Southampton Foundation NHS Trust, Southampton, UK.

^6^Global Health Research Institute, University of Southampton, Southampton, UK.

