# Supplementary material for: Pneumococcal vaccine impacts on the population genomics of non-typeable Haemophilus influenzae
Source: Microb Genom. 2018 Aug 6;4(9):e000209. doi: 10.1099/mgen.0.000209 (PMC6202451; doi:10.1099/mgen.0.000209)
Supplement: Supplementary File 1 [file mgen-4-209-s001.pdf]

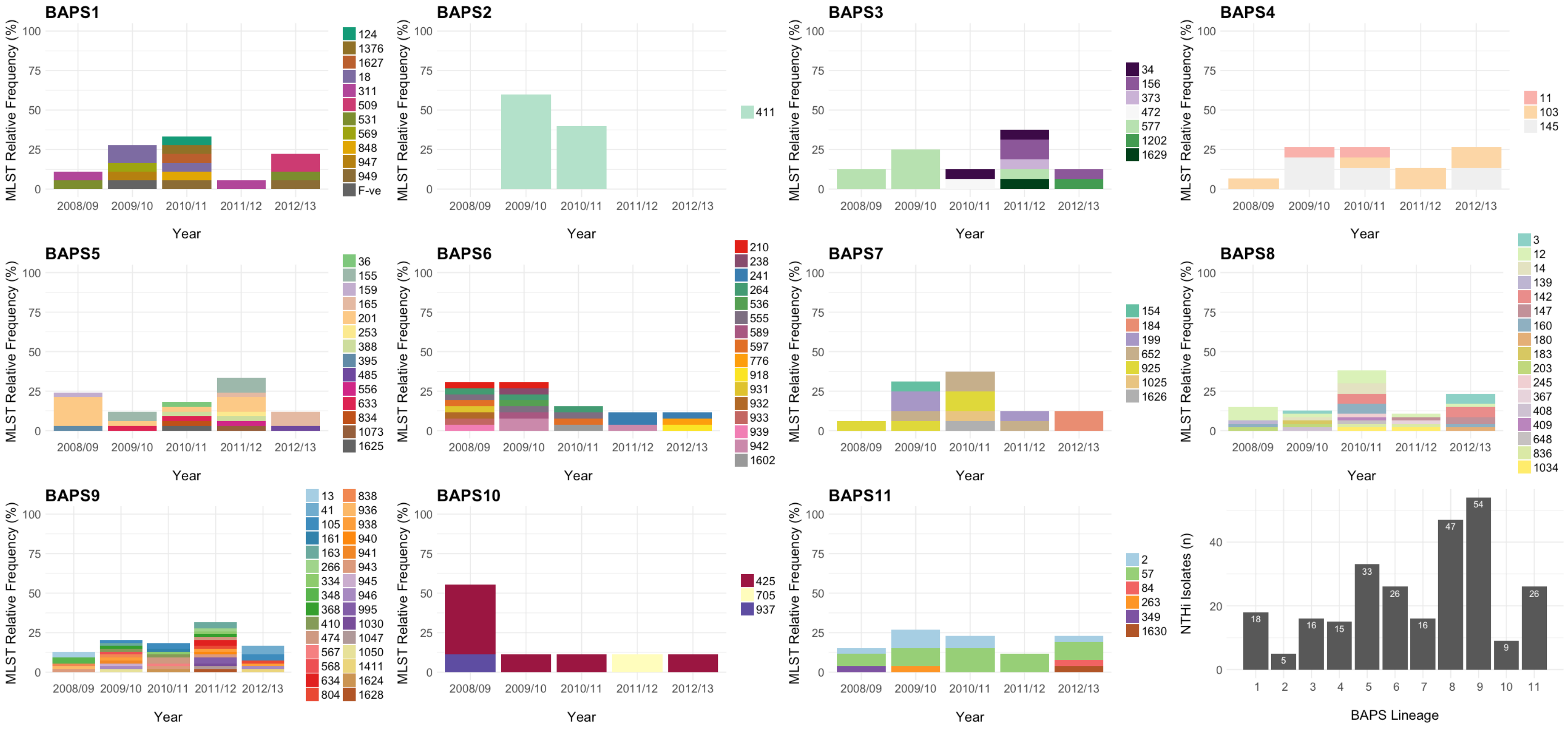

Supplementary Figure 1: Intra-lineage diversity of MLSTs in NTHi. The distribution of each MLST uniquely associated with a hierBAPS lineage (BAPS1-11) is shown. The cumulative isolate number for each lineage for all five years is also given in the bottom right plot.

Lineage	P/θ	δ (bp)	v	r/m	*p* (r/m)
1	0.854	88	0.046	3.47	0.0002*
2	0.184	3226	0.020	11.93	0.0521
3	0.831	892	0.031	23.11	0.8204
4	0.550	1343	0.039	28.74	0.9860
5	0.848	782	0.030	19.56	0.5440
6	0.904	641	0.027	15.51	0.2088
7	0.727	495	0.030	10.64	0.0276*
8	0.809	730	0.031	18.31	0.4312
9	0.534	277	0.031	4.65	0.0005*
10	1.103	1449	0.036	56.83	<0.0001*
11	0.819	692	0.030	17.07	0.3240
Mean	0.742	965	0.031	19.075	-
95% CI	0.771-0.713	865-1064	0.031-0.032	17.38-20.77	-

Supplementary Table 1: Extent of recombination identified in the eleven NTHi lineages. Output from ClonalFrame ML is shown. R/θ = ratio of recombination to mutation, δ = the mean length of recombination imports, ν = average divergence of imports, r/m = the relative effect of recombination to mutation. Higher r/m values indicate the change in rate at which a nucleotide is likely to be altered due to recombination compared to de novo mutation. Lineages 1 and 9 had significantly lower r/m compared to the population mean whereas lineage 10 was significantly higher (*p* values based on z test of population means).
